# The role of insurance status in molecular testing for thyroid nodules with indeterminate cytology

**DOI:** 10.1210/jendso/bvag179

**Published:** 2026-08-05

**Authors:** Kelvin Memeh, Daniel Chopyk, Bryan An, Stephanie Paras, Harold W Davis, Vennila Padmanaban, Barbra S Miller, John E Phay, Bridget A Oppong, Jesse J Plascak, Caitlin E Hackett, Samantha M Ruff, Priya H Dedhia

**Affiliations:** Division of Surgical Oncology, Department of Surgery, Ohio State University Comprehensive Cancer Center and Ohio State University Wexner Medical Center, Columbus, OH 43210, USA; Division of Surgical Oncology, Department of Surgery, Ohio State University Comprehensive Cancer Center and Ohio State University Wexner Medical Center, Columbus, OH 43210, USA; The Ohio State University College of Medicine, Columbus, OH 43210, USA; The Ohio State University College of Medicine, Columbus, OH 43210, USA; Division of Surgical Oncology, Department of Surgery, Ohio State University Comprehensive Cancer Center and Ohio State University Wexner Medical Center, Columbus, OH 43210, USA; Division of Surgical Oncology, Department of Surgery, Ohio State University Comprehensive Cancer Center and Ohio State University Wexner Medical Center, Columbus, OH 43210, USA; Division of Surgical Oncology, Department of Surgery, University of Arizona College of Medicine, Tucson, AZ 85721, USA; Division of Surgical Oncology, Department of Surgery, Ohio State University Comprehensive Cancer Center and Ohio State University Wexner Medical Center, Columbus, OH 43210, USA; Division of Surgical Oncology, Department of Surgery, Ohio State University Comprehensive Cancer Center and Ohio State University Wexner Medical Center, Columbus, OH 43210, USA; The Division of Cancer Prevention and Control, Department of Internal Medicine, Ohio State University College of Medicine, Columbus, OH 43210, USA; Department of Radiology, Ohio State University Wexner Medical, Columbus, OH 43210, USA; Division of Surgical Oncology, Department of Surgery, Ohio State University Comprehensive Cancer Center and Ohio State University Wexner Medical Center, Columbus, OH 43210, USA; Division of Surgical Oncology, Department of Surgery, Ohio State University Comprehensive Cancer Center and Ohio State University Wexner Medical Center, Columbus, OH 43210, USA; Clinical & Translational Science Institute, The Ohio State University College of Medicine, Columbus, OH 43210, USA

**Keywords:** molecular testing, barriers to care, thyroid nodule, thyroid cancer, uninsured, Bethesda category

## Abstract

**Objective:**

The aim of the study was to evaluate the effect of health insurance status on rate of molecular testing (MT) and subsequent thyroidectomy in patients with indeterminate thyroid nodules in a real-world high-volume clinical setting.

**Methods:**

This is a single-center, retrospective study of patients who underwent fine-needle aspiration and had Bethesda III–IV cytology results between 2014 and 2022. We excluded patients with prior thyroid cancer, thyroidectomy, or incomplete insurance data. We utilized multivariable logistic regression to identify significant predictors of MT utilization and surgical intervention; specifically examining the role of insurance status and clinical symptoms.

**Results:**

A total of 448 patients were included (median age: 52 years, 79% women) with 79% of patients undergoing MT; with an associated 27-percentage-point (34% vs 61%, *P* < .001) reduction in thyroidectomy. Lack of MT was the strongest predictor of surgery (adjusted odds ratios [aOR]: 4.50, 95% confidence interval [CI]: 2.71-7.47, *P* < .001). However, uninsured/self-pay patients had 68% lower odds of MT (aOR: 0.32, 95% CI: 0.12-0.88, *P* = .026) and approximately 19-percentage-point higher thyroidectomy rate compared to insured patients (59% vs 40%). Thyroid-related compression symptoms were also significantly associated with increased rate of thyroidectomy (aOR: 1.86, *P* = .007) and an appropriate reduction in MT utilization (aOR: 0.62, *P* = .046).

**Conclusion:**

Despite high MT penetration at an academic medical center, lack of health insurance poses a critical barrier to evidence-based risk stratification leading to higher rates of diagnostic lobectomy. While symptoms appropriately drove surgical management, expanding affordable MT access may mitigate barriers to care for patients with thyroid nodules.

The optimal management of thyroid nodules requires careful risk stratification and assessment. Fine-needle aspiration (FNA) cytology is the mainstay of this process; however, up to 30% of cases yield an indeterminate result (Bethesda category III or IV) (1, 2). Historically, these indeterminate results often led to a high rate of potentially avoidable thyroidectomies—as the risk of malignancy for Bethesda III (AUS/FLUS) and IV (FN/SFN) categories is approximately 22% (range 13-30%) and 30% (range 23-34%), respectively, per the 2023 Bethesda System update (3). This practice exposes many patients to potentially unwarranted surgical risks.

To mitigate overtreatment of thyroid nodules, genomic classifiers (molecular testing, MT) were introduced as an adjunct to FNA cytology. Since their endorsement by the American Thyroid Association in 2015 (1), multiple studies have demonstrated that MT can reduce thyroidectomy rates, though the magnitude of reduction varies by study design—from modest effects in population-level analyses (4) to surgical avoidance rates of 50-69% across platforms in individual-level and meta-analytic studies (5-11).

However, much of this evidence is drawn from insured patient cohorts and often presumes a simplified, linear care pathway. As MT has moved from controlled trials to real-world use, there are practical considerations about equitable access, determinants of testing adoption, and the clinical trajectory of patients who fall outside the standard “testing-to-surgery” model. Three gaps are salient. First, existing studies commonly aggregate uninsured patients with publicly insured cohorts, obscuring their unique challenges and clinical outcomes. Second, the predominant study design in the literature simplifies the clinical decision-making process into a binary choice—testing vs surgery—without accounting for patients who receive neither and may be lost to follow-up. Third, the intersectionality of clinical symptoms and socioeconomic factors is seldom explicitly modeled despite their influence on care decisions.

To address these gaps, our study examines MT utilization patterns at a high-penetration center, where utilization exceeds 75% for Bethesda III–IV cytology. The high overall MT utilization at our center reduces the likelihood that low MT uptake among uninsured patients reflects institutional reluctance or low provider familiarity. Our study offers insights into strategies to mitigate barriers to care and enhance thyroid nodule management in clinical practice.

## Methods

### Study setting and patient selection

We performed a single-center, retrospective analysis of patients who underwent FNA for thyroid nodule(s) at a tertiary, high-volume thyroid practice. Due to the retrospective nature of the study, the requirement of informed consent was waived and the study approved by the local Institutional Review Board (protocol #2023H-0234). We identified all FNA biopsies of thyroid nodules performed between January 1, 2014 and December 31, 2022, that yielded Bethesda III (Atypia of Undetermined Significance/Follicular Lesion of Undetermined Significance) or Bethesda IV (Follicular Neoplasm/Suspicious for Follicular Neoplasm) cytology. Exclusion criteria were prior thyroid malignancy or thyroidectomy before the index FNA, incomplete demographic or insurance data, and age <18 years. The decision to pursue MT (12), surgery, or observation was based on physician judgment and patient preference. FNA samples for cytology and MT were obtained under ultrasound guidance using 23-, 25-, or 27-gauge needles. MT was performed using commercial genomic classifier (Afirma platform, Veracyte, Inc. South San Francisco, CA). All FNA cytology specimens were interpreted locally at our institution by experienced thyroid cytopathologists; MT was performed on a separate dedicated FNA pass sent directly to Veracyte for Afirma genomic classifier analysis. Cytopathology interpretation and MT were therefore performed independently. Of note, no patients in our cohort underwent repeat FNA as an alternative management pathway; clinical trajectories following indeterminate cytology consisted of MT alone with surveillance, MT followed by thyroidectomy, thyroidectomy without MT, or neither MT nor thyroidectomy. For patients who underwent surgery, histopathology was reviewed by head and neck pathologists.

### Data collection and variables

Sociodemographic variables (age, sex, and self-reported race/ethnicity), insurance status, and residential ZIP code were abstracted from the electronic health record. Insurance status was categorized as private (managed care and commercial payer), government insurance (Medicare or Medicaid), or self-pay/uninsured. Patients’ ZIP codes were used to derive Social Deprivation Index (SDI)—a validated measure, estimated from annual American Community Survey sociodemographic data, transformed into national percentiles, and linked to each patient by residential zip code and date of visit encounter (higher values indicating greater social deprivation) (13, 14). Clinical variables, including compressive symptoms or laboratory indicator of hyperthyroidism at presentation (binary), ultrasound characteristics, molecular test order and result, and whether patient underwent thyroidectomy were also retrieved for analysis. Nodule size and ACR TI-RADS sonographic features were extracted from the free-text ultrasound reports using a rule-based natural language processing algorithm (regular-expression and keyword pattern matching); when both an algorithm-computed and a radiologist-documented TI-RADS category were available for a nodule, the higher (more suspicious) level was adopted. The extraction approach, level adjudication, and cohort data flow are detailed in the Supplementary Methods (Fig. S1 and Table S1) (15).

The primary outcomes of this study were the predictors of MT of the index FNA specimen, and rates of thyroidectomy (lobectomy or total thyroidectomy) within 12 months of the index clinic visit. A 12-month window was chosen to capture thyroidectomy related to the index FNA evaluation while minimizing inclusion of surgeries prompted by subsequent clinical changes.

### Statistical analysis

Continuous data are summarized as mean ± SD (or median and IQR when non-normal) and compared with *t*-tests or Mann–Whitney *U*, as appropriate. Categorical data are presented as counts (%) and compared with χ^2^ or Fisher tests. Variables associated with either outcome on univariable analysis at *P* ≤ .05, or prespecified as clinically relevant based on prior literature on MT utilization, were entered into multivariable logistic regression models as covariates to adjust for confounding. Insurance status, thyroid symptoms, and SDI were treated as confounders of the MT–thyroidectomy relationship rather than as competing exposures, and the resulting estimates should be interpreted as adjusted associations rather than independent causal contributions. Because MT informs surgical decision-making, the model evaluating thyroidectomy rates included MT status to account for potential differences in risk stratification. Further, in the thyroidectomy model, MT was operationalized as a binary variable (informative MT vs no/noninformative MT). MT results were classified as informative (benign or suspicious) vs noninformative (inconclusive, insufficient specimen, or parathyroid tissue). Noninformative results were grouped with no-MT because they did not yield a definitive risk-stratifying result.

Given the typical clinical pathway follows a sequential causal structure (ultrasound features → FNA decision → Bethesda cytology → MT decision → surgical decision), and our cohort is conditioned on Bethesda III/IV cytology, which is downstream of the ultrasound-guided FNA decision, ultrasound risk features (specifically nodule size and ACR TI-RADS category) were not included as covariates in the primary multivariable models for MT and thyroidectomy. We recognized, however, that nodule size or TI-RADS category could each plausibly influence the decision to operate or to pursue MT through pathways that are not captured in our dataset; we therefore added them to both primary models in sequential sensitivity analyses.

Patients with missing data for thyroidectomy or MT status were excluded from the multivariable analysis. Model results are reported as adjusted odds ratios (aORs) with 95% confidence intervals (CIs). Analyses were performed with Stata 18 (StataCorp, College Station, TX); two-sided *P* < .05 denoted significance.

## Results

### Baseline characteristics

A total of 448 unique patients met the study inclusion criteria, of which 79% were females. Most patients had some form of health insurance (private 55%, government 40%), while 3.8% (n = 17) were self-pay/uninsured. Overall, 79% of patients underwent MT, and 41% of the total cohort proceeded to thyroidectomy. Baseline characteristics stratified by surgical and MT status are summarized in Table 1. The mean diameter of the index nodule was 23.7 mm (median, 20 mm; interquartile range [IQR], 14-31 mm; Table 1). The size distribution across all scored nodules and the completeness of size and TI-RADS data are shown in Fig. S2 and Table S2 (15). Of the 448-patient cohort, 184 underwent a thyroidectomy (median age: 47 years, IQR: 37-58). We could not ascertain thyroidectomy status in 3 patients, who were therefore excluded from the multivariable analysis.

**Table 1:** Baseline characteristics of the study cohort, stratified by receipt of thyroidectomy and by molecular testing

Characteristic	Thyroidectomy			Molecular testing		
	No (n = 261)	Yes (n = 184)	*P* value	No (n = 95)	Yes (n = 353)	*P* value
Age, y	54.7 (14.6)	47.4 (13.6)	**<.001**	50.9 (15.3)	51.9 (14.6)	.53
Age category			**<.001**			.15
<45 y	70 (26.8%)	82 (44.6%)		39 (41.1%)	114 (32.3%)	
45-64 y	109 (41.8%)	78 (42.4%)		32 (33.7%)	156 (44.2%)	
≥65 y	82 (31.4%)	24 (13.0%)		24 (25.3%)	83 (23.5%)	
Sex			.44			.51
Female	202 (77.4%)	148 (80.4%)		77 (81.1%)	275 (77.9%)	
Male	59 (22.6%)	36 (19.6%)		18 (18.9%)	78 (22.1%)	
Race			.43			.43
Asian	8 (3.1%)	4 (2.2%)		2 (2.1%)	10 (2.8%)	
Black	42 (16.1%)	38 (20.7%)		22 (23.2%)	58 (16.4%)	
Caucasian	199 (76.2%)	130 (70.7%)		65 (68.4%)	267 (75.6%)	
Other/unknown	12 (4.6%)	12 (6.5%)		6 (6.3%)	18 (5.1%)	
Ethnicity			.33			.29
Hispanic	6 (2.3%)	3 (1.6%)		0 (0.0%)	9 (2.5%)	
Non-Hispanic	247 (94.6%)	179 (97.3%)		93 (97.9%)	336 (95.2%)	
Unknown	8 (3.1%)	2 (1.1%)		2 (2.1%)	8 (2.3%)	
Marital status			.50			.073
Married	152 (58.2%)	113 (61.4%)		49 (51.6%)	218 (61.8%)	
Single	109 (41.8%)	71 (38.6%)		46 (48.4%)	135 (38.2%)	
Employment status			**<.001**			.89
Employed or self-employed	96 (36.8%)	88 (47.8%)		36 (37.9%)	149 (42.2%)	
Not employed	50 (19.2%)	38 (20.7%)		22 (23.2%)	66 (18.7%)	
Part-time employed	13 (5.0%)	19 (10.3%)		6 (6.3%)	26 (7.4%)	
Retired	96 (36.8%)	33 (17.9%)		29 (30.5%)	101 (28.6%)	
Student	3 (1.1%)	4 (2.2%)		1 (1.1%)	7 (2.0%)	
Unknown	3 (1.1%)	2 (1.1%)		1 (1.1%)	4 (1.1%)	
Thyroid symptoms			**<.001**			**.015**
No	198 (75.9%)	109 (59.2%)		56 (58.9%)	254 (72.0%)	
Yes	63 (24.1%)	75 (40.8%)		39 (41.1%)	99 (28.0%)	
Nodule size, mm	22.2 (12.4)	25.9 (19.2)	**.034**	27.6 (23.8)	22.7 (12.8)	**.025**
Nodule size category			.25			.46
0-1 cm	25 (13.1%)	15 (11.2%)		6 (9.4%)	36 (13.6%)	
1.1-2 cm	75 (39.3%)	50 (37.3%)		21 (32.8%)	104 (39.4%)	
2.1-4 cm	77 (40.3%)	50 (37.3%)		29 (45.3%)	98 (37.1%)	
>4 cm	14 (7.3%)	19 (14.2%)		8 (12.5%)	26 (9.8%)	
TI-RADS category			.87			.23
TR3	87 (33.3%)	63 (34.2%)		31 (32.6%)	119 (33.7%)	
TR4	107 (41.0%)	71 (38.6%)		33 (34.7%)	148 (41.9%)	
TR5+	67 (25.7%)	50 (27.2%)		31 (32.6%)	86 (24.4%)	
Insurance status			.13			**.040**
Insured	253 (97.3%)	170 (94.4%)		87 (92.6%)	339 (97.1%)	
Uninsured	7 (2.7%)	10 (5.6%)		7 (7.4%)	10 (2.9%)	
Insurance type			**.034**			**.043**
Government	116 (44.4%)	63 (34.2%)		45 (47.4%)	135 (38.2%)	
Private	137 (52.5%)	107 (58.2%)		42 (44.2%)	204 (57.8%)	
Self-pay	7 (2.7%)	10 (5.4%)		7 (7.4%)	10 (2.8%)	
Unknown	1 (0.4%)	4 (2.2%)		1 (1.1%)	4 (1.1%)	
Primary language			.11			.78
English	251 (96.2%)	170 (92.4%)		91 (95.8%)	333 (94.3%)	
Not English	9 (3.4%)	14 (7.6%)		4 (4.2%)	19 (5.4%)	
Unknown	1 (0.4%)	0 (0.0%)		0 (0.0%)	1 (0.3%)	
Charlson–Deyo score	2.5 (2.4)	1.6 (2.1)	**<.001**	1.8 (2.2)	2.2 (2.4)	.16
Social Deprivation Index	40.4 (31.4)	46.9 (30.8)	**.034**	48.5 (32.2)	41.5 (30.8)	.054
Population	31 735 (17 525)	33 003 (17 740)	.46	30 902 (18 228)	32 638 (17 488)	.40

Data are presented as mean (SD) for continuous variables and No. (%) for categorical variables. Boldface indicates *P* < .05. Category percentages are column percentages computed among nonmissing observations and may not sum to the stratum total; for several variables (eg, nodule-size category, TI-RADS), the denominator is smaller than the column N because of missing data. *P* values reflect bivariate association tests between each characteristic and the stratifying variable; specify the exact tests used (eg, t test or Wilcoxon rank-sum for continuous variables; χ^2^ or Fisher exact for categorical variables) in the Methods.

Abbreviations: SD, standard deviation; TI-RADS, Thyroid Imaging Reporting and Data System.

Three hundred and fifty-three patients (79%) had MT (median age: 52 years, IQR: 41-64), resulting in a benign (63%), suspicious for malignancy (29%), or noninformative/parathyroid (7%) results. Of those who had an informative MT result (N = 327), 34% underwent thyroidectomy compared to 61% in the no/noninformative MT group (*P* < .001). Among the 223 patients who had benign MT, 13% subsequently underwent thyroidectomy—compared to a 61% thyroidectomy rate in the nontested group (*P* < .001).

Of 17 uninsured patients in the entire cohort, 59% underwent thyroidectomy compared to 40% of the insured patients. In both the uninsured and insured cohort, a similar proportion of patients did not undergo either MT or surgery: 6% (N = 1) vs 7% (N = 29), respectively. Among the 184 patients who underwent thyroidectomy, malignancy rates on final surgical pathology were comparable between insured and uninsured patients (34.7% vs 30.0%; Table 2). Noninvasive follicular thyroid neoplasm with papillary-like nuclear features was identified in 6 (3.5%) insured patients and none of the uninsured patients (Table 2).

**Table 2:** Final surgical pathology of resected thyroid nodules by insurance status (N = 184)

Final pathology	Insured	Uninsured	Total
	(n = 170)	(n = 10)	(N = 184*^a^*)
Malignant, n (%)	59 (34.7)	3 (30.0)	64
Benign, n (%)	98 (57.6)	7 (70.0)	107
NIFTP, n (%)	6 (3.5)	0 (0.0)	6
Missing/unavailable, n (%)	7 (4.1)	0 (0.0)	7

Abbreviation: NIFTP = noninvasive follicular thyroid neoplasm with papillary-like nuclear features.

^*a*^Total includes 4 patients with missing insurance data (2 malignant, 2 benign). Column percentages are calculated within each insurance group. NIFTP is reported separately from benign and malignant categories per the 2017 WHO reclassification.

### Predictors of thyroidectomy

**Figure 1:**
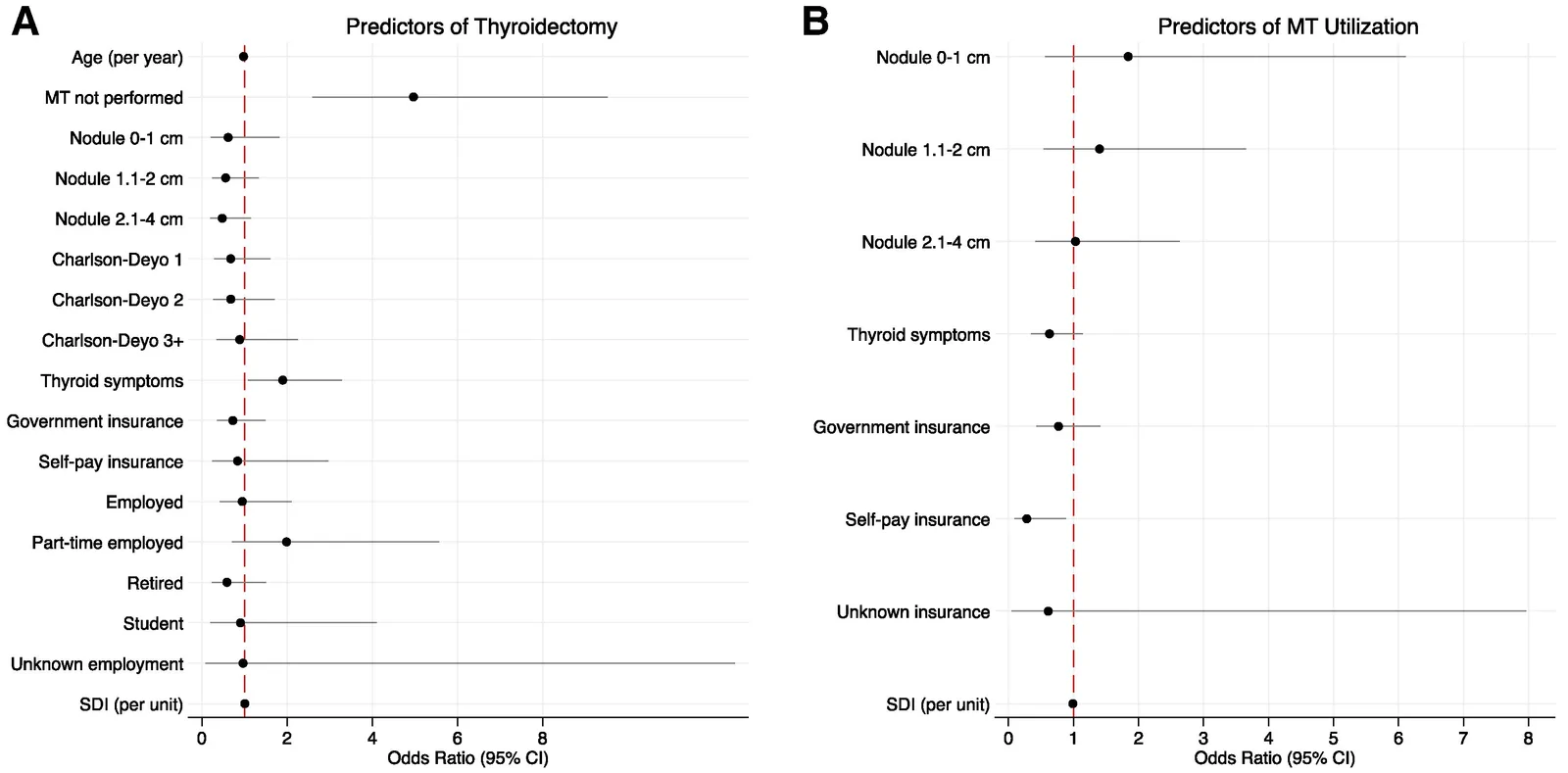
Forest plots of multivariable logistic regression analyses to predict thyroidectomy and molecular testing (MT) utilization. (A) Predictors of thyroidectomy within 12 months of index FNA. Lack of MT was the strongest predictor of surgery, while thyroid-related symptoms also increased surgical likelihood. Other variables, including comorbidity score, insurance category, and social deprivation index (SDI), were not significant. (B) Predictors of MT utilization. Self-pay/uninsured status and presence of thyroid-related symptoms were associated with reduced MT use, whereas government insurance and SDI were not significant predictors. Odds ratios are shown as points, with horizontal lines representing 95% confidence intervals. The vertical red dashed line indicates the null value (OR = 1.0).

On multivariable analysis, not undergoing MT was the strongest predictor of thyroidectomy (aOR: 4.50, 95% CI: 2.71-7.47, *P* < .001), corresponding to an approximately 4.5-fold higher odds of undergoing surgery. In the same model, thyroid-related symptoms was also associated with higher odds of undergoing thyroidectomy (aOR: 1.86, *P* = .007). Notably, insurance status was not a significant predictor of thyroidectomy after adjusting for MT utilization and other covariates; and patient age, employment status, and SDI were likewise not significant (Table 3, Fig. 1A). In sequential sensitivity models that added nodule size and then ACR TI-RADS category, neither was independently associated with thyroidectomy and the findings above were unchanged (Table S3) (15).

**Table 3:** Multivariable logistic regression of factors associated with thyroidectomy

Characteristic	aOR	95% CI	*P* value
Age at diagnosis, per year	0.98	0.95-1.00	.09
Molecular testing			
No	**4.50**	2.71-7.47	**<.001**
Yes	*1 [Reference]*		
Charlson–Deyo score			
1	1.14	0.54-2.41	.74
2	0.79	0.36-1.70	.54
3	1.31	0.62-2.77	.48
0	*1 [Reference]*		
Thyroid symptoms			
Yes	**1.86**	1.19-2.92	**.007**
No	*1 [Reference]*		
Insurance			
Government	0.69	0.40-1.21	.20
Self-pay	1.10	0.37-3.28	.86
Unknown	*Omitted (no observations)*		
Private/commercial	*1 [Reference]*		
Employment status			
Employed/self-employed	1.07	0.57-2.02	.83
Part-time employed	2.11	0.91-4.93	.08
Retired	0.64	0.30-1.35	.24
Student	1.23	0.27-5.61	.79
Unknown	1.11	0.19-6.32	.91
Unemployed	*1 [Reference]*		
Social deprivation index, per unit	1.01	1.00-1.01	.08

Model: N = 432; Wald χ^2^(14) = 67.06, *P* < .001; pseudo R^2^ = 0.13; log pseudolikelihood = −254.00. Nodule size and ACR TI-RADS category were excluded because both are assigned upstream of the fine-needle aspiration that defines the cohort; sequential models adding them are shown in Table S6 (15). Robust (Huber–White) standard errors. Boldface indicates *P* < .05.

Abbreviations: aOR, adjusted odds ratio; CI, confidence interval.

### Predictors of molecular test utilization

Univariable analyses showed significant differences in SDI, insurance and symptoms status between patients who did and did not receive MT. In the multivariable model, self-pay/uninsured patients were significantly less likely to receive MT than private- or government-insured peers (aOR: 0.32, 95% CI: 0.12-0.88, *P* = .026), representing a 68% reduction in odds of receiving MT (Table 4, Fig. 1B). Similarly, patients with symptomatic nodules were less likely to undergo MT (aOR: 0.62, *P* = .046). Interestingly, neighborhood SDI was not a significant predictor (Table 4, Fig. 1B) after adjusting for insurance status, suggesting that individual insurance coverage—rather than area-level socioeconomic factors—was the primary barrier to MT access. Government insurance (Medicare/Medicaid) was not significantly different from private insurance in predicting MT utilization (Table 4, Fig. 1B). The association of self-pay/uninsured with reduced molecular test utilization remained statistically robust to the sequential inclusion of nodule size and ACR TI-RADS category to the model; and neither sonographic feature was independently associated with MT (Table S4) (15).

**Table 4:** Multivariable logistic regression of factors associated with molecular testing

Characteristic	aOR	95% CI	*P* value
Thyroid symptoms			
No	*1 [Reference]*		—
Yes	**0.62**	0.38-0.99	**.046**
Insurance			
Private/Managed care	*1 [Reference]*		—
Government	0.69	0.43-1.13	.14
Self-pay/uninsured	**0.32**	0.12-0.88	**.026**
Unknown	0.77	0.08-7.63	.82
Social Deprivation Index (per unit)	0.99	0.99-1.00	.12

Boldface indicates *P* < .05.

Abbreviations: aOR, adjusted odds ratio; CI, confidence interval.

### Impact of MT on surgery

Among patients with informative MT results (N = 327), only 34% underwent thyroidectomy compared to 61% in the no-MT or noninformative MT group—a clinically significant 27-percentage-point reduction (*P* < .001). This translates to MT preventing surgery in approximately 1 in 4 patients who would have otherwise undergone diagnostic lobectomy. Over a mean 24-month follow-up period, none of the 194 patients with MT-benign nodules who did not have surgery developed clinical or ultrasound features concerning for malignancies.

When stratified by symptom status, asymptomatic patients (n = 310) had higher MT utilization (81.9% vs 71.7%, *P* = .015), lower thyroidectomy rates (35.5% vs 54.3%, *P* < .001), and comparable malignancy rates on final pathology (37.8% vs 33.3%, *P* = .38) relative to symptomatic patients (n = 138) (Table S5) (15). In a sensitivity analysis restricted to asymptomatic patients, lack of MT remained the strongest predictor of thyroidectomy (aOR: 4.39, 95% CI 2.01-9.56, *P* < .001), with no significant effect of insurance status (self-pay aOR: 0.65, *P* = .52; Table S6) (15). For MT utilization among asymptomatic patients, univariate analysis showed no significant association with insurance status (*P* = .57; Table S7) (15), though sparse cell sizes among uninsured asymptomatic patients (n = 9, of whom 2 did not receive MT) precluded stable multivariable adjustment.

It is worth noting that 30 patients underwent neither thyroidectomy nor MT during the study period, and their outcomes are summarized in Table S8 (15).

## Discussion

Our study reveals a clinically meaningful barriers to care in the management of thyroid nodules: despite 79% overall MT penetration at a high-volume academic center, uninsured patients were 68% less likely to receive MT. This barrier to risk stratification was associated with higher thyroidectomy rates among uninsured patients (59% vs 40% for insured patients), as surgery became necessary for definitive diagnosis. This finding is particularly concerning given that lack of MT was the strongest predictor of undergoing surgery (aOR: 4.50, *P* < .001), and that our short-term follow-up data are reassuring—none of the 194 MT-benign nodules developed malignancies over 24 months. Insurance-based barriers to MT thus drive uninsured patients toward diagnostic lobectomy rather than evidence-based surveillance.

Beyond identifying barriers to care, our study confirms that MT is associated with a substantial reduction in diagnostic lobectomy. Over a 24-month mean follow-up period, none of the patients with MT-benign nodules developed features concerning for malignancies, which is consistent with the high negative predictive value reported in prospective trials, although longer follow-up is required to fully establish the safety of MT-guided surveillance (9). Our study findings confirm that MT effectively replaces potentially low-yield diagnostic lobectomy with evidence-based surveillance for indeterminate and asymptomatic nodules. The observed 27-percentage-point reduction in thyroidectomy after MT use is consistent with findings from previous studies, including pooled surgical avoidance rates of 50-69% reported in a recent meta-analysis. While Huang et al reported that population-level thyroidectomy reductions may reflect broader de-escalation trends rather than MT adoption alone, our single-center data demonstrate a clear association between MT utilization and thyroidectomy avoidance at the individual patient level, consistent with evidence from meta-analysis and clinical trial (5, 8, 9).

We also observed that thyroid compressive symptoms were associated with a higher thyroidectomy rate, after adjustment for MT results, and with reduced odds of MT (aOR: 0.62, *P* = .046; Table 4). To assess whether ultrasound features could account for the observed associations, we entered nodule size and ACR TI-RADS category into both primary models in sequential sensitivity analyses. Neither was associated with either outcome, and the associations we report in the primary model were unchanged (Tables S3 and S4) (15). These sonographic features were not included in the primary models because they are assigned upstream of the FNA that defines this cohort. Within a population already selected on indeterminate cytology, the decisions to operate and to test are primarily driven by the cytologic result and by clinical and system factors rather than by the sonographic features that prompted the biopsy. Notably, malignancy rates on final pathology were comparable between symptomatic and asymptomatic patients (33.3% vs 37.8%, *P* = .38; Table S5) (15), supporting that thyroidectomy in symptomatic patients was driven by therapeutic indications rather than higher cancer suspicion. These data reveal that clinical practice at our center reflects a de facto symptom-stratified approach in which symptomatic patients were less likely to undergo MT and more likely to proceed to thyroidectomy, consistent with appropriate clinical decision-making. However, patients with mild symptoms may still reasonably pursue MT to inform extent of resection or support nonoperative management.

Our analysis isolated a specific barrier to care: self-pay and uninsured patients were significantly less likely to receive MT than their commercially insured counterparts, despite care occurring in a high-penetration center. Within the thyroid literature, insurance-related barriers to care in timing of surgical treatment as well as appropriate follow-up among uninsured and Medicaid patients are well-established (16-26). Our findings are consistent with these patterns; however, our study introduces restricted MT access in thyroid nodule management as a potential mechanism. Literature specifically addressing MT access barrier in thyroid nodule management remains limited. However, several studies have reported income and insurance-related differences in thyroid care: A 2023 claims-based study by Ginzberg et al reported that Medicaid patients not only present with more locally advanced thyroid cancer but are also less likely to receive timely surgical treatment and subsequent radioactive iodine therapy (18). When uninsured patients must choose between the costs of evidence-based MT and surveillance vs proceed directly to surgery, they may opt for surgery to avoid ongoing testing expenses and follow-up burden. Our data support this pattern: uninsured patients were less likely to receive MT (aOR: 0.32, *P* = .026) and had higher unadjusted thyroidectomy rates compared with insured patients—even after adjusting for nodule characteristics. This trend of socioeconomically disadvantaged patients receiving more invasive treatment while having less access to diagnostic testing extends across other thyroid conditions. Jin et al. found that over 94% of patients who underwent thyroidectomy for Graves’ disease, rather than standard medical management, were either uninsured or of low-income status (19). Compounding these access barriers, the majority of thyroid surgeries in the United States are performed by low-volume surgeons (those performing ≤25 cases annually), who have significantly higher complication rates than high-volume surgeons (20). Patients from socioeconomically disadvantaged communities are disproportionately more likely to be treated by low-volume surgeons (21), placing uninsured patients at dual risk: they are both more likely to undergo thyroidectomy and more likely to experience surgical complications. Furthermore, Song et al. reported that patients from high poverty areas were less likely to receive any follow-up care for thyroid nodules, even when initial FNA results were Bethesda III or higher (22).

These access barriers extend beyond thyroid cancer to other oncology settings. Yabroff et al found that oncologists’ decisions to order genomic testing are influenced by patient insurance coverage and anticipated out-of-pocket costs (23). This effect translates to a disappointing clinical impact: Dibiase et al demonstrated that low-income patients have reduced access to genome-informed treatment, resulting in poorer survival outcomes (24). These broad trends in oncology are also exemplified in nonsmall cell lung cancer, where uninsured and Medicaid patients are less likely to undergo biomarker testing or receive targeted therapy compared with privately insured patients (25, 26).

Altogether, our findings have both clinical and upstream policy implications. First, our data demonstrate that MT is associated with a substantial reduction in potentially unnecessary diagnostic lobectomy in approximately 1 in 4 patients with indeterminate nodules, with reassuring short-term follow-up data. Second, symptoms appropriately stratify patients: symptomatic nodules warrant surgery regardless of MT results, suggesting that clinical decision tools should discourage MT in this population to avoid unnecessary costs—unless MT results could alter the extent of thyroidectomy (eg, lobectomy vs total). Third, and most critically, lack of insurance creates a modifiable barrier to MT access that perpetuates health inequities. Notably, malignancy rates on final pathology were comparable between insured and uninsured patients (34.7% vs 30.0%; Table 2), suggesting that uninsured patients are not being selected for surgery based on higher clinical suspicion but rather are proceeding to diagnostic lobectomy due to the inability to access MT for risk stratification. Given that lack of insurance may preclude patients from the benefits of MT risk stratification, targeted interventions are urgently needed. Institutional strategies could include streamlining enrollment in manufacturer-sponsored financial assistance programs or subsidizing MT costs for uninsured patients.

### Strengths and limitations

Our study has several strengths, including a prospectively maintained database, standardized thyroid nodule work-up, high MT penetration (which reduces selection bias), and multivariable modeling. However, its limitations include its retrospective, single-institution design, small sample size of uninsured individuals, use of ZIP code as an area-level proxy for neighborhood socioeconomic deprivation, and potential for residual confounding by surgeon preference or unmeasured socioeconomic factors. Additionally, the high MT penetration rate at our institution may limit generalization to centers with lower adoption rates. Additionally, our study exclusively utilized the Afirma Genomic Sequencing Classifier (Veracyte). Two other commercially available molecular assays—ThyroSeq (CBLPath/University of Pittsburgh) and ThyGenX/ThyraMIR (Interpace Diagnostics)—are in clinical use in the United States, each with different performance characteristics and cost structures, and our findings may not be directly generalizable to centers using alternative platforms. We also note that insurance-based barriers to MT are particularly relevant in healthcare systems without universal coverage; in countries with publicly funded healthcare, the mechanism identified here may be mitigated, though other access barriers (eg, geographic, institutional adoption) may persist. Lastly, a longer follow-up period is required to exclude late false negatives among MT-benign nodules, and our 24-month observation period is insufficient to make definitive claims about the long-term safety of MT-guided surveillance.

## Conclusion

Molecular genomic classifiers remain an effective strategy for reducing unnecessary diagnostic thyroidectomies with reassuring short-term oncologic outcomes in real-world high-volume practice. However, their use is influenced by 2 key factors. Nodule-related symptoms appropriately drive thyroidectomy regardless of MT results indicating that MT is most valuable in asymptomatic indeterminate nodules. In contrast, financial barriers—through lack of insurance- limit access to MT and may constitute a barrier to thyroid nodule management. These findings suggest that expanding access to MT could improve equity in thyroid nodule management, while a symptom-stratified approach may help avoid unnecessary MT in patients who clearly need surgery. Larger, multi-institutional studies are needed to confirm our results and guide policy intervention for equitable thyroid care. Specifically, targeted interventions are needed to reduce barriers to MT access.

## Data Availability

Restrictions apply to the availability of some, or all data generated or analyzed during this study to preserve patient confidentiality or because they were used under license. The corresponding author will on request detail the restrictions and any conditions under which access to some data may be provided.

## References

[1] Haugen BR, Alexander EK, Bible KC, et al 2015 American Thyroid Association management guidelines for adult patients with thyroid nodules and differentiated thyroid cancer: the American Thyroid Association guidelines task force on thyroid nodules and differentiated thyroid cancer. Thyroid. 2016;26(1):1‐133. 26462967 10.1089/thy.2015.0020 PMC4739132

[2] Durante C, Hegedüs L, Czarniecka A, et al 2023 European Thyroid Association clinical practice guidelines for thyroid nodule management. Eur Thyroid J. 2023;12(5):e230067. 37358008 10.1530/ETJ-23-0067 PMC10448590

[3] Ali SZ, Baloch ZW, Cochand-Priollet B, Schmitt FC, Vielh P, VanderLaan PA. The 2023 Bethesda system for reporting thyroid cytopathology. Thyroid. 2023;33(9):1039‐1044. 37427847 10.1089/thy.2023.0141

[4] Huang Y, Chan SJ, Wright JD, et al Does the adoption of molecular testing cause decreased thyroidectomy rates in a national cohort? A quasiexperimental study of high- versus low-adoption states. Thyroid. 2024;34(3):388‐398. 38251649 10.1089/thy.2023.0651

[5] Chowdhury R, Hier J, Payne KE, et al Impact of molecular testing on surgical decision-making in indeterminate thyroid nodules: a systematic review and meta-analysis of recent advancements. Cancers (Basel). 2025;17(7):1156. 40227659 10.3390/cancers17071156 PMC11987950

[6] Steward DL, Carty SE, Sippel RS, et al Performance of a multigene genomic classifier in thyroid nodules with indeterminate cytology. JAMA Oncol. 2019;5(2):204‐212. 30419129 10.1001/jamaoncol.2018.4616 PMC6439562

[7] Borowczyk M, Szczepanek-Parulska E, Olejarz M, et al Evaluation of 167 gene expression classifier and ThyroSeq v2 diagnostic accuracy in indeterminate thyroid nodules. Endocr Pathol. 2019;30(1):8‐15. 30591992 10.1007/s12022-018-9560-5

[8] Livhits MJ, Zhu CY, Kuo EJ, et al Effectiveness of molecular testing techniques for diagnosis of indeterminate thyroid nodules: a randomized clinical trial. JAMA Oncol. 2021;7(1):70‐77. 33300952 10.1001/jamaoncol.2020.5935 PMC7729582

[9] Vargas-Salas S, Martínez JR, Urra S, et al Genetic testing for indeterminate thyroid cytology: review and meta-analysis. Endocr Relat Cancer. 2018;25(3):R163‐R177. 29255094 10.1530/ERC-17-0405 PMC5799829

[10] Krane JF, Cibas ES, Endo M, et al Afirma Xpression Atlas for thyroid nodules and thyroid cancer metastases: insights to inform clinical decision-making from a fine-needle aspiration sample. Cancer Cytopathol. 2020;128(7):452‐459. 32543766 10.1002/cncy.22300 PMC7384066

[11] Nikiforova MN, Mercurio S, Wald AI, et al Analytical performance of the ThyroSeq v3 genomic classifier for cancer diagnosis in thyroid nodules. Cancer. 2018;124(8):1682‐1690. 29345728 10.1002/cncr.31245 PMC5891361

[12] Patel KN, Angell TE, Babiarz J, et al Performance of a genomic sequencing classifier for the preoperative diagnosis of cytologically indeterminate thyroid nodules. JAMA Surg. 2018;153(9):817‐824. 29799911 10.1001/jamasurg.2018.1153 PMC6583881

[13] Rebechi M, Kohlschmidt J, Mrozek K, et al Association of social deprivation with survival in younger adult patients with acute myeloid leukemia. Blood Adv. 2023;7(15):4019‐4023. 37196637 10.1182/bloodadvances.2022009325 PMC10425796

[14] Butler DC, Petterson S, Phillips RL, Bazemore AW. Measures of social deprivation that predict health care access and need within a rational area of primary care service delivery. Health Serv Res. 2013;48(2 Pt 1):539‐559. 22816561 10.1111/j.1475-6773.2012.01449.x PMC3626349

[15] Memeh K, Chopyk DM, An B, et al Supplementary Material from Memeh et al., The role of insurance status in molecular testing for thyroid nodules with indeterminate cytology. Deposited in Figshare, 1 August 2026. doi:10.6084/m9.figshare.33135494 PMC13504264 42643595

[16] Chen DW, Yeh MW. Disparities in thyroid care. Endocrinol Metab Clin North Am. 2022;51(2):229‐241. 35662439 10.1016/j.ecl.2021.11.017 PMC9174593

[17] Ginzberg SP, Soegaard Ballester JM, Wirtalla CJ, et al Racial and ethnic disparities in appropriate thyroid cancer treatment before and after the 2015 American Thyroid Association guidelines. Ann Surg Oncol. 2023;30(5):2928–22937. 36749501 10.1245/s10434-023-13158-3 PMC13247833

[18] Ginzberg SP, Soegaard Ballester JM, Wirtalla CJ, et al Insurance-based disparities in guideline-concordant thyroid cancer care in the era of de-escalation. J Surg Res. 2023;289:211‐219. 37141704 10.1016/j.jss.2023.03.046 PMC10229451

[19] Jin J, Sandoval V, Lawless ME, et al Disparity in the management of Graves disease observed at an urban county hospital. Am J Surg. 2012;204(2):199‐202. 22317948 10.1016/j.amjsurg.2011.10.010 PMC3623456

[20] Adam MA, Thomas S, Youngwirth L, et al Is there a minimum number of thyroidectomies a surgeon should perform to optimize patient outcomes? Ann Surg. 2017;265(2):402‐407. 28059969 10.1097/SLA.0000000000001688

[21] Al-Qurayshi Z, Randolph GW, Srivastav S, et al Outcomes in thyroid surgery are affected by racial, economic, and healthcare system demographics. Laryngoscope. 2016;126(9):2194‐2199. 27139800 10.1002/lary.25871

[22] Song Z, Balachandra S, Wu C, et al Loss of follow-up for thyroid nodules in patients living in poverty. Endocr Pract. 2025;31(2):169‐175. 39551186 10.1016/j.eprac.2024.11.005 PMC12320506

[23] Yabroff KR, Shi KS, Zhao J, et al Importance of patient health insurance coverage and out-of-pocket costs for genomic testing in oncologists’ treatment decisions. JCO Oncol Pract. 2024;20(3):429‐437. 38194620 10.1200/OP.23.00153 PMC11742181

[24] DiBiase JF, Scharnetzki E, Edelman E, et al Socioeconomic and urban-rural disparities in genome-matched treatment receipt and survival after genomic tumor testing. JNCI Cancer Spectr. 2024;8(5):pkae090. 39312685 10.1093/jncics/pkae090 PMC11483106

[25] Bruno DS, Hess LM, Li X, Su EW, Patel M. Disparities in biomarker testing and clinical trial enrollment among patients with lung, breast, or colorectal cancers in the United States. JCO Precis Oncol. 2022;6:e2100427. 35737912 10.1200/PO.21.00427

[26] Roberts TJ, Kehl KL, Brooks GA, et al Practice-level variation in molecular testing and use of targeted therapy for patients with non-small cell lung cancer and colorectal cancer. JAMA Netw Open. 2023;6(4):e2310809. 37115543 10.1001/jamanetworkopen.2023.10809 PMC10148196

